# Parishin E from ginger-processed *Gastrodia elata* Bl. alleviates rheumatoid arthritis by regulating histone 3 lactylation at H3K18la and H3K27la sites

**DOI:** 10.3389/fphar.2025.1682504

**Published:** 2025-10-28

**Authors:** Xinyue Liu, Yijing Pan, Chenxi Deng, Meiliang Zhu, Dongmei Guo, Jiaqin Wu, Fan Feng, Lianhong Pan, Chunli Wang, Kang Xu

**Affiliations:** ^1^ Hubei Provincial Engineering Technology Research Center for Chinese Medicine Processing, School of Pharmacy, Hubei University of Chinese Medicine, Wuhan, China; ^2^ Hubei University of Chinese Medicine, Wuhan, China; ^3^ School of Laboratory Medicine, Hubei University of Chinese Medicine, Wuhan, China; ^4^ Chongqing Key Laboratory of Development and Utilization of Genuine Medicinal Materials in Three Gorges Reservoir Area, Chongqing Engineering Research Center of Antitumor Natural Drugs, Chongqing Three Gorges Medical College, Chongqing, China

**Keywords:** rheumatoid arthritis, ginger-processedtreated Gastrodia elata Bl., Gastrodia elata Bl., non-targeted metabolomics, glycolysis, H3 histone lactylation

## Abstract

**Background:**

This study investigated the natural small molecule drug Parishin E (PE) derived from the orchid plant *Gastrodia elata* Bl. (GEB). We evaluated the therapeutic effects of ginger-processed *Gastrodia elata* Bl. (G-GEB) on rheumatoid arthritis (RA) and focused on paracine E to elucidate its potential regulatory mechanisms.

**Methods:**

An Sprague-Dawley rat model of rheumatoid arthritis was constructed to evaluate the pharmacological effects of G-GEB. Plant non-targeted metabolomics, serum non-targeted metabolomics, and RAW264.7 inflammation models elucidate Parishin E as the core anti-inflammatory component of G-GEB. Subsequently, transcriptomic and metabolomic analyses were performed to elucidate the molecular signaling mechanism of Parishin E in the treatment of RA.

**Results:**

G-GEB significantly improves RA, with 363 active compounds identified by untargeted metabolomics. PE, its main active component, affects cell glycolysis by downregulating HK2 and LDHA to inhibit macrophage polarization. PE also shows anti-inflammatory properties by suppressing H3 lactylation at H3K18la and H3K27la.

**Conclusion:**

PE in G-GEB exerts an RA ameliostatic effect by inhibiting macrophage polarization by regulating cellular glycolysis and by inhibiting the emulsylation modification of histone H3 sites, specifically H3K18la and H3K27la. Therefore, PE is a promising drug candidate for the development of RA treatments.

## Introduction

G-GEB demonstrates antispasmodic, neuroregulatory (e.g., sedative and hypotensive), as well as microcirculation-enhancing and anti-inflammatory properties (Su et al., 2023). Its chemical components are complex, and the main components, including Gastrodin, Parishin, and Polysaccharides, have been demonstrated to have potential mechanisms in anti-inflammatory, sedative, and immune-regulatory effects (Li Y. et al., 2025; Zhu et al., 2024).

G-GEB is prepared by stir-frying GEB moistened with ginger juice. Rich in active components such as gingerols, ginger juice not only facilitates the release of bioactive constituents from GEB but also promotes peripheral circulation, modulates thermoregulation, reduces edema, and exerts analgesic effects. Consequently, it enhances therapeutic efficacy against conditions such as rheumatic arthralgia and dizziness. However, systematic studies on the active components of G-GEB, as well as the chemical and pharmacological differences between G-GEB and GEB, remain relatively limited (Li S. et al., 2025). Non-targeted phyto-metabolomics can reveal the chemical composition changes between G-GEB and GEB, offering significant insight into their pharmacological activity transformation (Pan et al., 2024).

Rheumatoid arthritis is a chronic inflammatory autoimmune disease characterized by symmetrical polyarthritis, affecting primarily the synovium, cartilage, and bone, resulting in joint damage and dysfunction (Guo et al., 2024; Liu et al., 2025; Bao et al., 2024; Ke et al., 2025). RAW264.7 cells play a crucial role in the pathogenesis of RA (Liu et al., 2017; Zeng et al., 2025; Wu et al., 2020), These cells can be activated by LPS to mimic the inflammatory response of RA *in vitro*, leading to the secretion of large amounts of pro-inflammatory factors, including TNF-α, IL-6, TGF-β, and MMPs (Luo et al., 2024), Inflammatory signaling promotes the expression of glycolysis-related genes, including GLUT1, HK2, PKM2, and LDHA (Wei et al., 2024; Wang et al., 2020a), The upregulation of these genes enhances glucose uptake and lactate production, thereby supporting the secretion of pro-inflammatory factors (Zhao et al., 2024).

Inflammation and metabolism are closely related, and metabolic disorders may serve as a key factor in the development and progression of inflammatory diseases (Tan et al., 2022; Wang et al., 2020b), Inflammation is the core pathological mechanism of RA. Lactylation, as an important protein post-translational modification, regulates metabolic enzyme activity, participates in feedback regulation processes, enhances HIF-1α transcriptional activity, and promotes glycolysis and lactate generation (Zhang and Zhang, 2024; Xu et al., 2024). Lactylation relies on lactate and is written by acetyltransferases and erased by deacetylases, dynamically regulating protein function and gene expression (Feng et al., 2024; Yang et al., 2024).

This study employed Complete Freund’s Adjuvant (CFA)-induced Adjuvant Arthritis (AIA) rat models to compare GEB and GEB pharmacologically. Using non-targeted phyto-metabolomics, it analyzed their chemical differences and explored the basis for GEB’s enhanced efficacy. The LUOMICS™ TCM omics platform identified G-GEB’s blood-absorbed components, revealing key component PE, which significantly increased in content and contributed to G-GEB’s improved efficacy. By downregulating key glycolytic enzymes, PE reduced LPS-induced *in vitro* inflammation in RAW264.7 cells, decreasing histone lactylation at lysine residues 18 and 27 via SIRT6 regulation. This finding provides a chemical basis for G-GEB’s enhanced efficacy.

## Materials and methods

### Reagents and materials

Parishin E (CAS No:952068-57-4, CFN93115), Methotrexate (MTX) (CAS No:498-02-2, CFN60179) were purchased from ProSpec Bio-Tech Co., Ltd. (Chengdu, China). All reagents had a purity of >98%. Lipopolysaccharide (LPS) was purchased from Thermo Fisher Scientific (United States). The goat anti-mouse and anti-rabbit secondary antibodies were obtained from Solarbio Group (Beijing, China). Complete Freund’s Adjuvant was purchased from Sigma-Aldrich (St. Louis, MO, United States).

### Sample Preparation

GEB material was purchased from Chengdu Kangmei Pharmaceutical Co., Ltd., batch number: 240511231. It was identified by Professor Liu Yanju of Hubei University of Chinese Medicine as the dried rhizome of *Gastrodia elata* Bl., a plant of the Orchidaceae family, with the voucher sample number 202409-S1. The G-GEB herbal slices were processed by the Processing Laboratory of Hubei University of Chinese Medicine.

Processing method: GEB was stir-fried with ginger juice following this protocol: moistening for 2 h, then stir-frying with 20 mL ginger juice at 150 °C for 7 min. Voucher specimens were deposited at Hubei University of Chinese Medicine (Wuhan, China).

100 g GEB/G-GEB powder was extracted twice via reflux with 50% ethanol (8 × volume) for 1 h each. Combined filtrates were rotary-evaporated at 60 °C to 200 mL, followed by ethanol removal in a 100 °C water bath. The residue was lyophilized and stored at −80 °C. Extraction yields were 15.3% (GEB) and 19.18% (G-GEB).

### Experimental animals

Sprague Dawley (SD) rats (180 ± 20 g) were procured from the Experimental Animal Center of Three Gorges University (License SCXK (E) 2022-0012). They were maintained on a 12-h light-dark cycle (artificial light) at 25 °C, with *ad libitum* access to water and standard chow. Animals were acclimated for 7 days in hygienic group housing before experimentation. This study was approved by the Experimental Animal Ethics Committee of Hubei University of Chinese Medicine (Animal Ethics and Welfare Approval Number: HUCMS2024110605).

### Construction and treatment of CFA-Induced Adjuvant Arthritis (AIA) in rats

Post-acclimation, 42 male rats were randomized into seven groups by body weight (n = 6): Control, AIA model, MTX, High-/Low-dose GEB, and High-/Low-dose G-GEB groups. Except for Controls, all rats received 0.1 mL CFA subcutaneously in the left hind paw on day 0 (Zarei et al., 2024). On day 7 post-immunization, MTX group rats were administered 0.2 mg/kg methotrexate orally, three times per week (Shen et al., 2022).

According to the Pharmacopoeia of the People’s Republic of China (2020 edition), Volume I, the adult clinical dosage is 3–10 g, as specified under the GEB monograph. Adult low and high doses were set at 5 g and 10 g. For a 60 kg adult, high doses of GEB and G-GEB are 25.5 mg/kg and 31.97 mg/kg. The rat equivalent dose is 6.3 × higher. GEB-H rats received 160.65 mg/kg/day *Gastrodia elata* Bl. ethanol extract; GEB-L rats received 80.32 mg/kg/day. G-GEB-H rats received 201.39 mg/kg/day ginger-processed extract; G-GEB-L rats received 100.70 mg/kg/day (He et al., 2020). Control rats received equal volumes of normal saline daily. All treatments were given once daily for 28 days.

### Therapeutic efficacy and evaluation of G-GEB in treating complete CFA-Induced AIA in rats

Post-CFA sensitization, body weight and paw swelling were recorded every 4 days. From day 7, three blinded observers assessed the arthritis score (AI) every 4 days. The scoring system: 0 = no redness/swelling; 1 = mild toe joint redness/swelling; 2 = toe joint and toe redness/swelling; 3 = redness/swelling below ankle; 4 = toe joint, toe, and ankle redness/swelling (Pan et al., 2025). On the experiment’s final day, overnight fasted rats were anesthetized with 40 mg/kg sodium pentobarbital via intraperitoneal injection (Shen et al., 2022). The Thymus and spleen were excised, weighed, and rinsed with PBS. Organ indices were calculated as organ-to-body weight ratios. Rat left hind ankle joints were fixed in 4% paraformaldehyde for 48 h, then decalcified in 10% EDTA for 40 days.

### Histopathological analysis

Paraffin-embedded samples were sectioned at 4 μm thickness and stained with HE and Safranin-O Fast-Green to assess tissue lesions. Histopathological scores were blindly evaluated by three independent histologists using the OARSI scoring system to quantify cartilage degeneration in the ankle joint.

### Non-targeted plant metabolomics for identifying differential chemical components

#### Sample pretreatment

Weigh six samples (three per group) of GEB/G-GEB (100 mg each) into 1.5 mL centrifuge tubes. Add two steel beads and 100 μL of water (with 4 μg/mL internal standards) to each. Precool at −40 °C for 2 min, grind at 60 Hz for 2 min, sonicate in ice-water for 30 min, then return to −40 °C for 2 h. After centrifugation (12000 rpm, 4 °C, 20 min), transfer supernatant to LC vials (Mengliang et al., 2022).

#### Analytical conditions

The analytical instrument was a Waters ACQUITY UPLC I-Class plus coupled to a Thermo-QE-HF high-resolution mass spectrometer. An ACQUITY-UPLC-HSS-T3 column (100 mm × 2.1 mm, 1.8 μm) was used at 45 °C. The mobile phase consisted of solvent A (0.1% formic acid in water) and solvent B (acetonitrile) at a 0.35 mL/min flow rate. The injection volume was 5 μL.

### Pharmacokinetic analysis of bioactive components in G-GEB

#### Plasma sample pretreatment

Twelve male SD rats (180 ± 20 g) were acclimatised for 7 days and then randomly divided into a blank control group and a G-GEB treatment group (n = 6). The blank control group was administered an equal volume of physiological saline, while the G-GEB treatment group was administered G-GEB ethanol extract at a dose of 201.39 mg/kg/day via gavage for 7 days. On day 6, the rats were fasted. On day 7, blood was collected using heparin sodium anticoagulant tubes 2 h after the final gavage. The blood was mixed and centrifuged (5000 rpm, 4 °C, 15 min), and the upper layer of plasma was collected and stored at −80 °C for later use.

Plasma (150 μL) was added to a 1.5 mL EP tube, followed by 450 μL protein precipitant (methanol-acetonitrile, 2:1, with 4 μg/mL internal standards). After vortexing (1 min) and sonication in ice-water (10 min), samples were incubated at −4 °C for 30 min. Centrifugation (12000 rpm, 4 °C, 10 min) was preceded by transferring 500 μL supernatant to an LC-MS vial and drying. Samples were reconstituted with 150 μL water-methanol-acetonitrile (1:2:1), vortexed (1 min), sonicated (3 min), and stored at −40 °C overnight. After re-centrifugation (12000 rpm, 4 °C, 10 min), 100 μL supernatant was transferred to an LC-MS vial with a footed insert for analysis (Sun et al., 2023).

### Preparation of G-GEB freeze-dried powder samples

Grind the sample in liquid nitrogen, transfer ∼100 mg to a 1.5 mL tube, add 1 mL water (4 μg/mL internal standards), vortex 1 min, and add steel beads. Pre-chill at −40 °C for 2 min, grind at 60 Hz for 2 min. Sonicate in ice-water for 60 min, then centrifuge (12000 rpm, 4 °C, 10 min). Dilute supernatant 2-fold with water (4 μg/mL internal standards), transfer 200 μL to an LC-MS vial for analysis.

### Analysis conditions

Analysis was performed using an ACQUITY UPLC I-Class HF system with a QE high-resolution mass spectrometer. Chromatography was performed using an ACQUITY UPLC HSS T3 column (100 mm × 2.1 mm, 1.8 μm) at 45 °C. The mobile phase was 0.1% formic acid in water (A) and acetonitrile (B) at 0.35 mL/min. The HESI ion source was used with DDA acquisition in full MS/dd-MS2 (Top 8) mode. The injection volume was 5 μL, and the PDA scan range was 210–400 nm.

### Appraisal process and standards

The identification of differential metabolites and blood components in this study primarily relied upon a self-constructed reference database (TCM database) containing information on over 5,000 standard reference materials for traditional Chinese medicine constituents. Compound identification strictly required complete matching of their retention times (RT) and MS/MS spectra with those in the reference database. For the few peaks that failed to match within the self-constructed database, supplementary matching was performed using public databases such as HMDB. This strategy, grounded in the proprietary reference database, maximises the accuracy and reliability of the identified constituents in both the original TCM formulae and the bioavailable components. Compound identification strictly adheres to a hierarchical validation protocol: retention time deviation from database standards must be within ±0.3 min; primary molecular weight deviation must be within 5 ppm; and measured MS2 spectra must align with standard MS2 spectra. Where secondary fragmentation peaks are absent, identification is confirmed by cross-referencing retention time and primary molecular weight deviation against standard comparisons.

### Cell culture

Mouse macrophage RAW 264.7 cells (species: mouse, *Mus musculus*; CVCL_0439; #CL-0190) purchased from Pricella (Wuhan, China). The cells were cultured in DMEM medium containing 10% foetal bovine serum (FBS; catalogue number 164210-50, Pricella), 100 U/mL penicillin, and 100 μg/mL streptomycin at 37 °C in a 5% CO_2_ incubator.

### Cell viability of RAW264.7 cells was detected by the CCK-8 method

The effect of PE on cell viability was assessed using the CCK-8 assay. RAW264.7A cells (5000 cells per well) were seeded into 96-well plates and exposed to different concentrations of PE (1, 2, 4, 8, 16, 32, 64, 128, 256 μM) for 24 h. Subsequently, 10 μL of CCK-8 reagent was added to each well, and the plates were incubated for 3 h. The absorbance (OD value) at 450 nm was measured for each well using a microplate reader (BioTek, United States).

### Western blotting assay

Cellular total and nuclear proteins were extracted using RIPA lysis buffer and the Beyotime Kit (Shanghai, China), respectively, with protein concentrations determined by BCA assay. Following SDS-PAGE electrophoresis, target proteins were transferred to PVDF membranes. Membranes were blocked with 5% skim milk in TBST for 2 h, incubated with primary antibodies overnight at 4 °C, and allowed to incubate with HRP-conjugated goat anti-mouse IgG (H + L) (1:5000, Cat#SE131, RRID:AB_2797595, Solarbio) or HRP-conjugated goat anti-rabbit IgG (H + L) (1:5000, Cat#SE134, RRID:AB_2797593, Solarbio) second antibodies at room temperature for 1 h with gentle agitation. After washing, proteins were visualized using ECL reagents and band densities quantified with ImageJ software.

Primary antibodies used were as follows: IL-6 (1: 1000, Proteintech, Cat# IL6-b, RRID:AB_3665394), TNF-α (1: 1000, ABclonal, Cat# A20851, RRID:AB_3669044), MMP1 (1: 1000, Boster Bio, Cat# PB0764, RRID: N/A), MMP9 (1: 1000, Boster Bio, Cat# BM4089, RRID: N/A), MMP13 (1: 1000, Abcam, Cat# ab315267, RRID:AB_3696043), GLUT1 (1:1000, WanLeiBio Cat# WL01163, RRID:AB_3675696), HK2 (1:1 000, Proteintech Cat# 22029-1-AP, RRID:AB_11182717), PKM2 (1: 1000, Proteintech Cat# 15822-1-AP, RRID:AB_1851537), LDHA (1: 1000, Beyotime, Cat# AF0216, RRID:N/A), LYS18 (1: 1000, PTM BIO Cat# PTM-158, RRID:AB_3678563), LYS27(1: 1000, PTM BIO Cat# PTM-160, RRID:AB_3697632), Pan Kla (1: 1000, PTM BIO Cat# PTM-1425, RRID:AB_2937057),β-Actin (1: 10000, Solarbio Cat# K101527P, RRID:AB_3697466), Histone H3 (1: 1000, PTM BIO Cat# PTM-312, RRID:AB_3711312), SIRT6 (1: 1000, ZenBio Cat# AR381408, RRID:N/A), HDAC1(1: 1000, ZenBio Cat# R24533, RRID:N/A), SIRT1 (1: 1000, Zen Bio Cat# R25721, RRID:AB_2921365), SIRT2 (1: 1000, Zen Bio Cat# R25722, RRID:N/A), SIRT7 (1: 1000, Proteintech Cat# 12994-1-AP, RRID:AB_10644276).

### Real-time polymerase chain reaction (RT-PCR)

Total RNA was extracted from RAW264.7 cells using TRIZOL reagent (Thermo Fisher Scientific Inc., United States) according to the manufacturer’s instructions. Reverse transcription was performed with 2 μL of RNA using a reverse transcription kit (Vazyme Biotech, China) (Wei et al., 2023). Gene expression was assessed by RT-PCR and expressed as fold change, normalized to β-Actin expression using the 2^−ΔΔCT^ method. The primers used were listed in Table 1.

**TABLE 1:** The primer sequences for mRNA in RT-PCR.

Primer name	Primer sequences
IL-6-Mouse	Sense: CTGCAAGTGCATCATCGTTGTTC Anti-sense: TACCACTTCACAAGTCGGAGGC
MMP3-Mouse	Sense: AGGAGGCAGCAGAGAACCTA Anti-sense: GCAGAAGCTCCATACCAGCA
TNF-α-Mouse	Sense: GCCATAGAACTGATGAGAGGGAG Anti-sense: GGTGCCTATGTCTCAGCCTCTT
TGF-β-Mouse	Sense: CTGTATCGTGGAACAGGCGA Anti-sense: TTCACGTCAAACGAGAGCCA
INOS-Mouse	Sense: GCGCTCTAGTGAAGCAAAGC Anti-sense: AGTGAAATCCGATGTGGCCT
LDHA-Mouse	Sense: AACTTGGCGCTCTACTTGCT Anti-sense: TAGCCGCCTGAGGACTTACT
GLUT1-Mouse	Sense: GCAGCAAGACCGATGAACAC Anti-sense: TAGCCGAACTGCAGTGATCC
HK2-Mouse	Sense: AGGCTACCCGGAGTTGTTCT Anti-sense: TTCTGTTCCAATCCGGCGAT
PKM2-Mouse	Sense: ACTCAACCTTTCAACTTTGCGG Anti-sense: ACATTTCCACCAAAGCCCACT
β-Actin-Mouse	Sense: CCAAGCAGGAGTACGACGAG Anti-sense: CTGCAACCACAGCACGATTG

### Immunofluorescence staining

RAW264.7 cells were seeded in 24-well plates and stimulated with LPS (1 μg/mL) or LPS + PE (32 μM) overnight. Cells were fixed/permeabilized with 4% PFA/0.1% Triton X-100, blocked with 5% BSA (37 °C, 30 min), and incubated with anti-CD86 (1:100) and anti-iNOS (1:100) primary antibodies (4 °C overnight). After washing (3× PBST), cells were incubated with Alexa Fluor^®^488 secondary antibodies (1:400, 1 h, dark), counterstained with DAPI, and mounted using an anti-fade reagent. Confocal microscopy was used to image five fields of view for fluorescence quantification.

### Transcriptome sequencing analysis(RNA-seq)

Samples with RNA integrity (RIN ≥ 7), 28S/18S ratio ≥ 0.7, and RNA purity (A260/280–2.0; A260/230–2.0) meeting the highest quality criteria were sent to BGI Genomics (Wuhan, China) Co., Ltd. for RNA-seq analysis using the BGISEQ-500 platform. The sequencing data were further analyzed using R (version 3.5.1) to identify differentially expressed genes and perform Gene Set Enrichment Analysis (GSEA) to identify enriched pathways (García-Espinosa et al., 2024).

### Gas chromatography-mass spectrometry analysis

Cells in 6-well plates were treated with LPS (1 μg/mL) and PE (32 μM) for 24 h. Cells were washed twice with 0.9% saline and ice-cold PBS, then transferred to ice. After adding 250 μL ice-cold methanol, cells underwent three liquid nitrogen freeze-thaw cycles and centrifugation. The supernatant was dried via nitrogen evaporation at 37 °C, then 80 μL methoxyamine in pyridine (20 mg/mL) was added. After 2 min centrifugation and 2 h at 37 °C, 80 μL BSTFA was added. Following another 2 min spin and 1 h at 80 °C, the supernatant was analyzed by Trace 1300 GC-MS. Differential metabolites were identified using TCM chromatographic fingerprints and MetaboAnalyst (https://www.metaboanalyst.ca/) (Lanqing et al., 2023).

### In-cell western assay

Intracellular protein detection was performed using the Cell Label 700 Staining ICW Kit (Gene Company, 926–42091). RAW264.7 cells in 96-well plates, cultured to 70% confluence and treated with PE (32 μM) for 48 h, were fixed with 4% PFA after washing thrice with PBS. After permeabilization, LI-COR blocking solution was applied for 1 h at room temperature. Primary antibodies were applied overnight, followed by goat anti-mouse secondaries for 1 h in the dark. Cells were washed five times with 0.1% Tween-20 in PBS, and fluorescence was detected via Odyssey imaging (Wang et al., 2025).

### Statistical analysis

Experiments were triplicated, and data are shown as mean ± SD. Data analysis was conducted using SPSS Statistics 19.0. One-way ANOVA and Tukey’s test compared groups, while Kruskal–Wallis H tested scores. *P* < 0.05 was statistically significant.

## Results

### G-GEB evaluation of pathological arthritis in AIA rats

**FIGURE 1:**
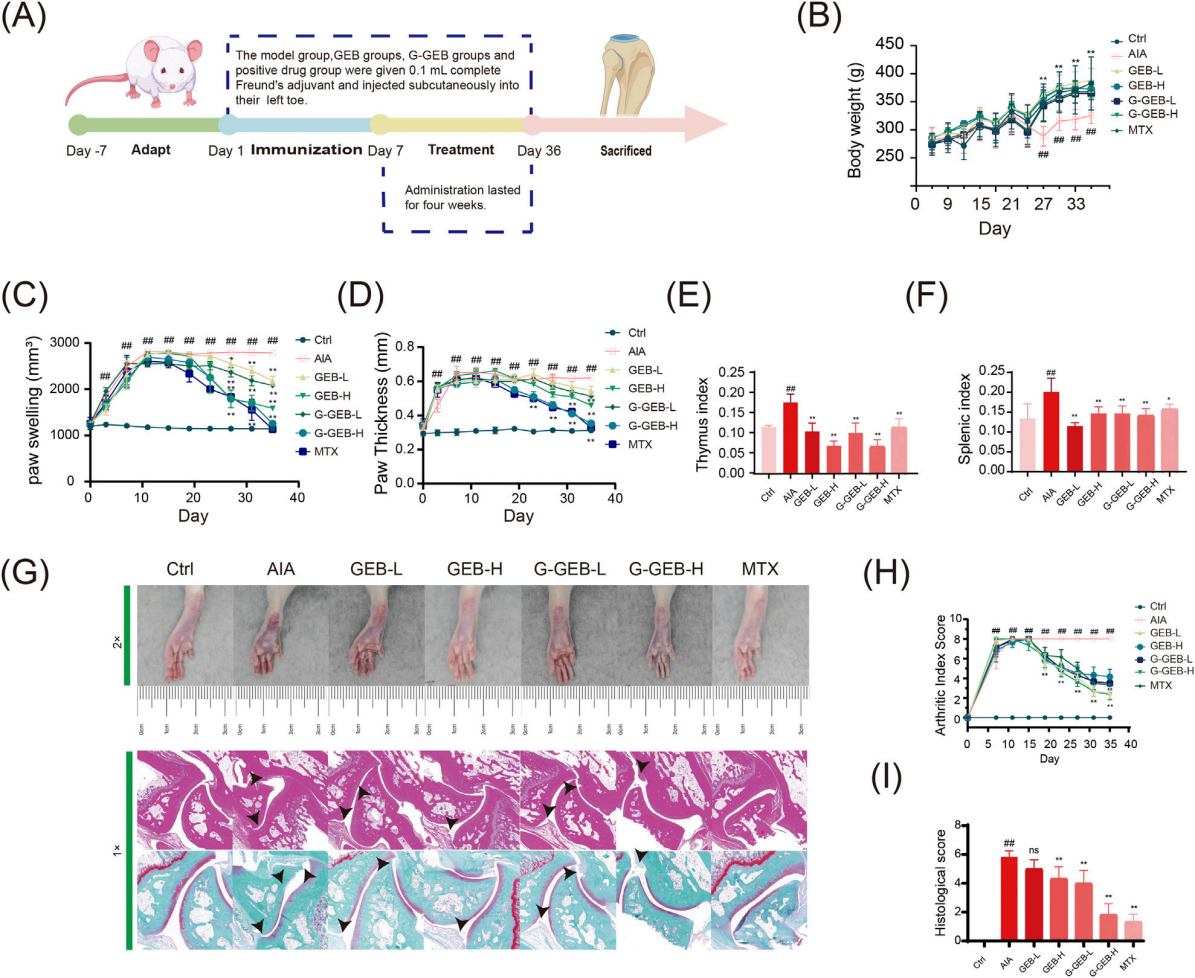
Pathological evaluation of arthritis in AIA rats treated by G-GEB. **(A)** Establishment of the AIA model and treatment regimen; **(B)** Changes in body weight of animals; **(C)** Record of toe swelling; **(D)** Changes in toe thickness; **(E,F)** Thymus and spleen index; **(G)** H&E and Safranin-O Fast-Green staining results; **(H)** Arthritis scoring; **(I)** Histopathological scoring. Data are the mean ± SD of 6 independent experiments. ^#^ *P* < 0.05, ^##^ *P* < 0.01, vs. Ctrl group; ^*^ *P* < 0.05, ^**^ *P* < 0.01, vs. AIA group; ns, not significant.

To evaluate the therapeutic efficacy of G-GEB on RA in rats, we designed and performed *in vivo* experiments (Figure 1A). Compared to the Control group, both AIA rats and treated rats exhibited slower body weight gain, with the most significant reduction observed in the AIA group (Figure 1B). AIA rats showed more toe swelling than Controls. GEB/G-GEB treatment reduced swelling, with G-GEB-H and GEB-H groups showing the greatest reduction by day 28 (Figure 1C). Throughout the experiment, toe thickness was measured, and the results were consistent with the changes in swelling (Figure 1D). AIA rats had higher thymus and spleen indices than Controls. CFA kept inflammation active, raising organ indices. GEB/G-GEB treatment normalized these indices (Figures 1E,F).

HE staining revealed inflammatory cell infiltration in synovial tissue across groups. The AIA group showed cartilage damage and irregular bone surfaces, while GEB/G-GEB treatment restored cartilage and improved cell regularity. Safranin O-fast green staining indicated fewer Safranin O-positive cells in AIA model rats, with cartilage wear and expanded bone tissue. Treatment groups had denser cartilage cell arrangements than the AIA group (Figure 1G). Bar charts of HE and Safranin O-fast green staining scores confirmed these results (Figure 1H). AIA model rats showed severe ankle joint damage. GEB/G-GEB treatment improved this damage, with G-GEB showing better therapeutic effects than GEB (Figure 1I).

## Untargeted metabolomics reveals key differences between GEB and G-GEB

**FIGURE 2:**
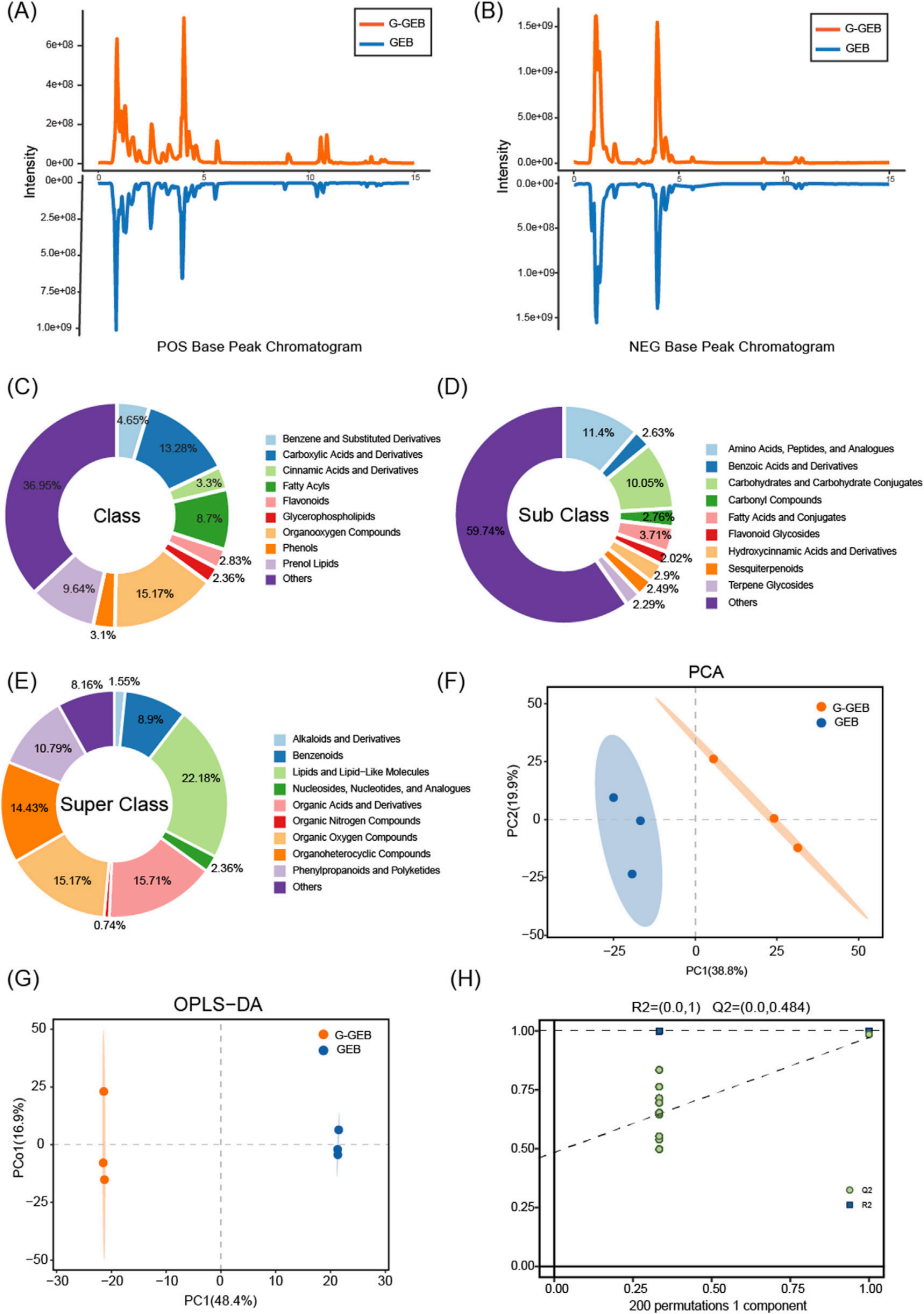
Chemical composition differences between GEB and G-GEB samples. **(A,B)** Comparison of base peak chromatograms of GEB and G-GEB in positive and negative ion modes; **(C–E)** Pie charts showing classification differences between GEB and G-GEB at class, subclass, and superclass levels; **(F,G)** PCA and OPLS-DA analyses showing clear separation between the GEB and G-GEB groups; **(H)** 200 permutation tests.

To systematically compare the metabolic composition differences between GEB and G-GEB, we performed a non-targeted metabolomics analysis. Comparison of the base peak chromatograms (BPC) showed significant differences in chromatographic peak intensity and retention time between the two groups in both positive and negative ion modes (Figures 2A,B), suggesting distinct overall metabolic profiles. Furthermore, the classification similarities and differences of the two groups of compounds at the class, subclass, and superclass levels were illustrated by pie charts (Figures 2C–E). Database - detected chemical components were divided into ten superclasses. Lipids and lipid-like molecules were the most abundant (22.18%), followed by organic acids and derivatives (15.71%).

PCA and OPLS-DA analyses showed clear separation between GEB and G-GEB groups (Figures 2F,G). To verify the reliability of the model, we conducted 200 permutation tests, and the results showed *R* ^2^ and Q^2^ values of 1 and 0.484, respectively (Figure 2H), indicating the model’s lack of overfitting and its good predictive ability and stability. The permutation test confirmed the analysis results’ reliability, showing the model accurately reflected the compound differences between GEB and G-GEB, thus accounting for their pharmacological differences.

### Identification of differential metabolites between the GEB and G-GEB groups

**FIGURE 3:**
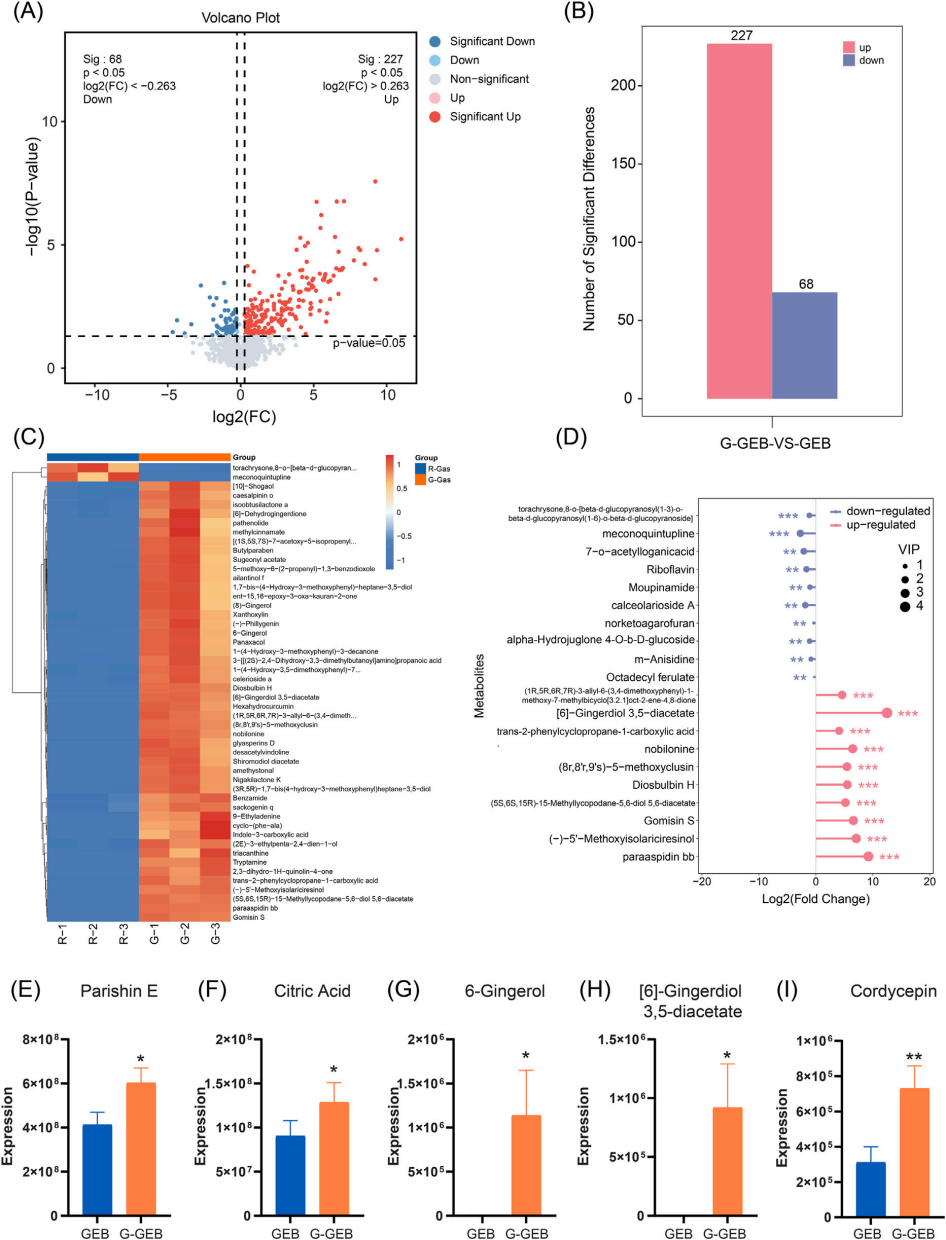
Identification of differential compounds between the GEB and G-GEB groups. **(A)** Volcano plot illustrated; **(B)** A bar chart as shown in figure B indicates alterations in the composition of chemical components between GEB and G-GEB; **(C)** The clustered heatmap in figure C reveals significant differences in the composition and proportion of chemical components between GEB and G-GEB; **(D)** Lollipop plot based on P-value criteria to count the top ten compounds with altered content between GEB and G-GEB; **(E–I)** Statistical analysis of peak areas is conducted for Parishin E, Citric Acid, 6-Gingerol, [6]-Gingerdiol 3.5-diacetate, and Cordycepin as depicted in figures **(E–I)**. Data are the mean ± SD of 6 independent experiments. ^*^ *P* < 0.05, ^**^ *P* < 0.01, vs. GEB group.

In order to gain deeper insights into the specific differences in chemical composition between GEB and G-GEB, this study conducted a differential analysis of compounds identified via non-targeted metabolomics. Volcano plot analysis revealed significant differences in compounds between the two groups (Figure 3A). Furthermore, the bar chart intuitively displayed the number distribution of differential compounds, with 227 significantly upregulated among the 363 differential compounds identified (Figure 3B). Supplementary Table S1 summarizes the top 50 significantly upregulated compounds. The heatmap illustrated the clustering relationship between the two sample groups, with colors shifting from blue to red to indicate changes in compound abundance, thereby emphasizing the significant differences between them (Figure 3C).

Findings-based lollipop charts presented the top 10 upregulated and downregulated differential compounds with the smallest P-values (Figure 3D). We analyzed compounds like Parishin E, Citric Acid, 6-Gingerol, [6]-Gingerdiol 3.5-diacetate, and Cordycepin, which showed significant post - processing increases. The analysis found six components with notably higher relative peak areas, especially Parishin and Citric Acid (Figures 3E–I).

### Plasma metabolomics analysis of original formula components

**FIGURE 4:**
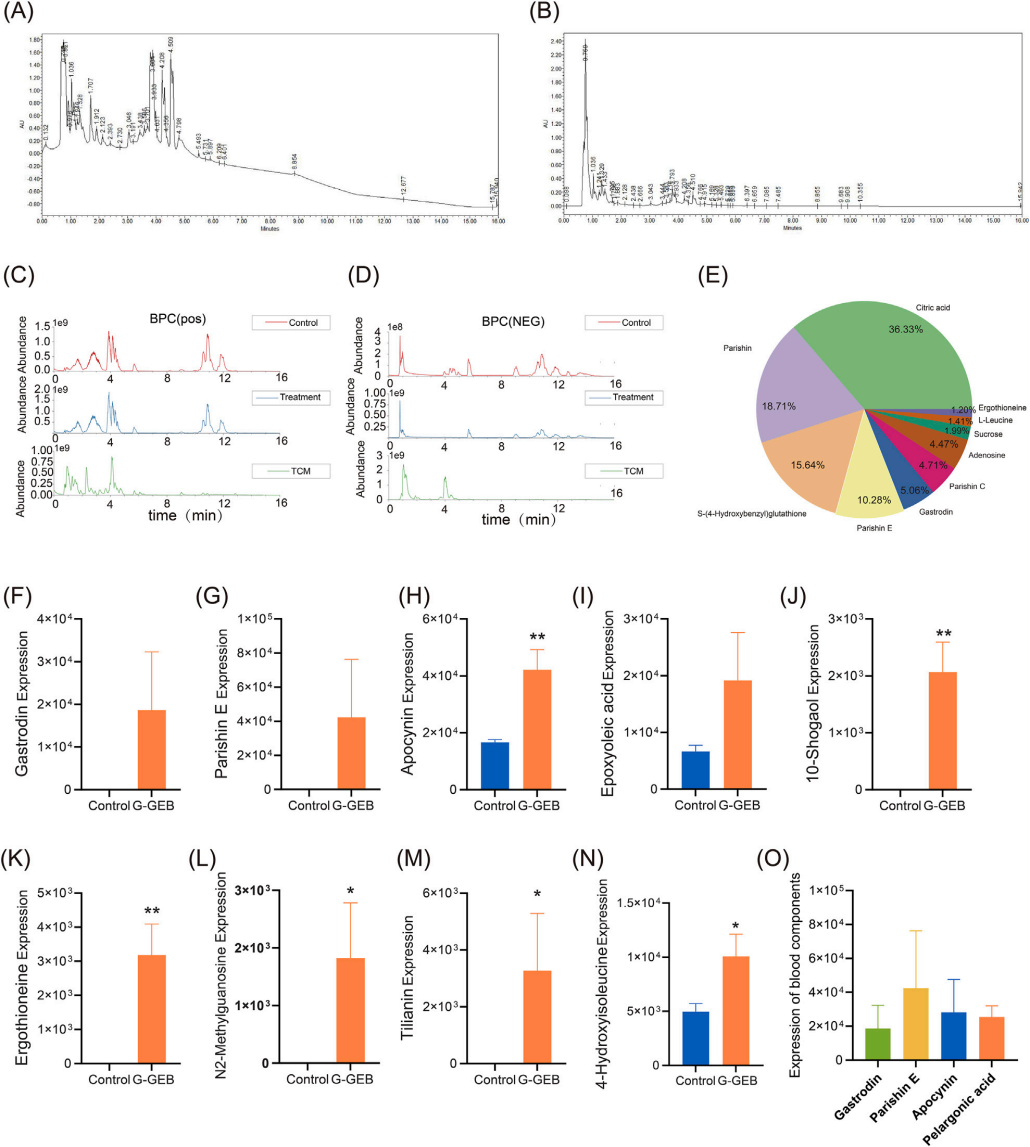
Identification of pharmacologically active blood components of G-GEB in animal models. **(A,B)** UV absorption spectra of plasma from rats administered G-GEB, measured at 210 nm and 254 nm; **(C,D)** Base peak chromatograms of blank plasma, drug-containing plasma, and the original herbal formula components under positive and negative ionization modes; **(E)** A pie chart displaying the top ten chemical components in the original formula; **(F–N)** Statistical charts comparing the relative peak areas of bioactive blood components between blank plasma and G-GEB - treated plasma; **(O)** highlights the four components with the highest plasma concentrations following G-GEB administration. Data are the mean ± SD of 6 independent experiments. ^*^ *P* < 0.05, ^**^ *P* < 0.01, vs. Control group.

To clarify the *in vivo* exposure of the original drug components in G-GEB, this study systematically analyzed the prototype compounds and their metabolites in plasma of normal rats after administration using plasma metabolomics. Strong UV absorption at 210 nm and 254 nm indicated the presence of various saponins and phenyl-containing compounds in G-GEB (Figures 4A,B). The base peak chromatograms in positive and negative ion modes are shown in Figures 4C,D. The top ten components by abundance were Citric Acid, Parishin, Parishin E, Gastrodin, Parishin C, Adenosine, Sucrose, Ergothioneine, L-leucine, and others (Figure 4E).

### Analysis of G-GEB metabolites in plasma

In plasma samples after treatment with G-GEB, a total of 33 metabolites were identified, including 15 prototype compounds and 18 metabolites. Through literature screening, we identified 9 compounds with reported good biological activity, including Gastrodin, Parishin E, Vanillylacetone, Rac-cis-9,10-epoxyoctadecanoic acid, 10-Gingerol, Ergothioneine, N2-Methylguanosine, Scoparin, and 4-Hydroxyisoleucine (Figures 4F–N).

By analyzing the database and comparing the original formula components with the plasma of the G-GEB-treated group, 11 chemical constituents were identified in both groups (Supplementary Table S2). Further quantitative analysis revealed that Gastrodin, Parishin E, Apocynin, and Nonanoic acid were the top four compounds by plasma concentration (Figure 4O).

### PE significantly inhibits LPS-induced inflammatory response in macrophage cells under *in vitro* conditions

**FIGURE 5:**
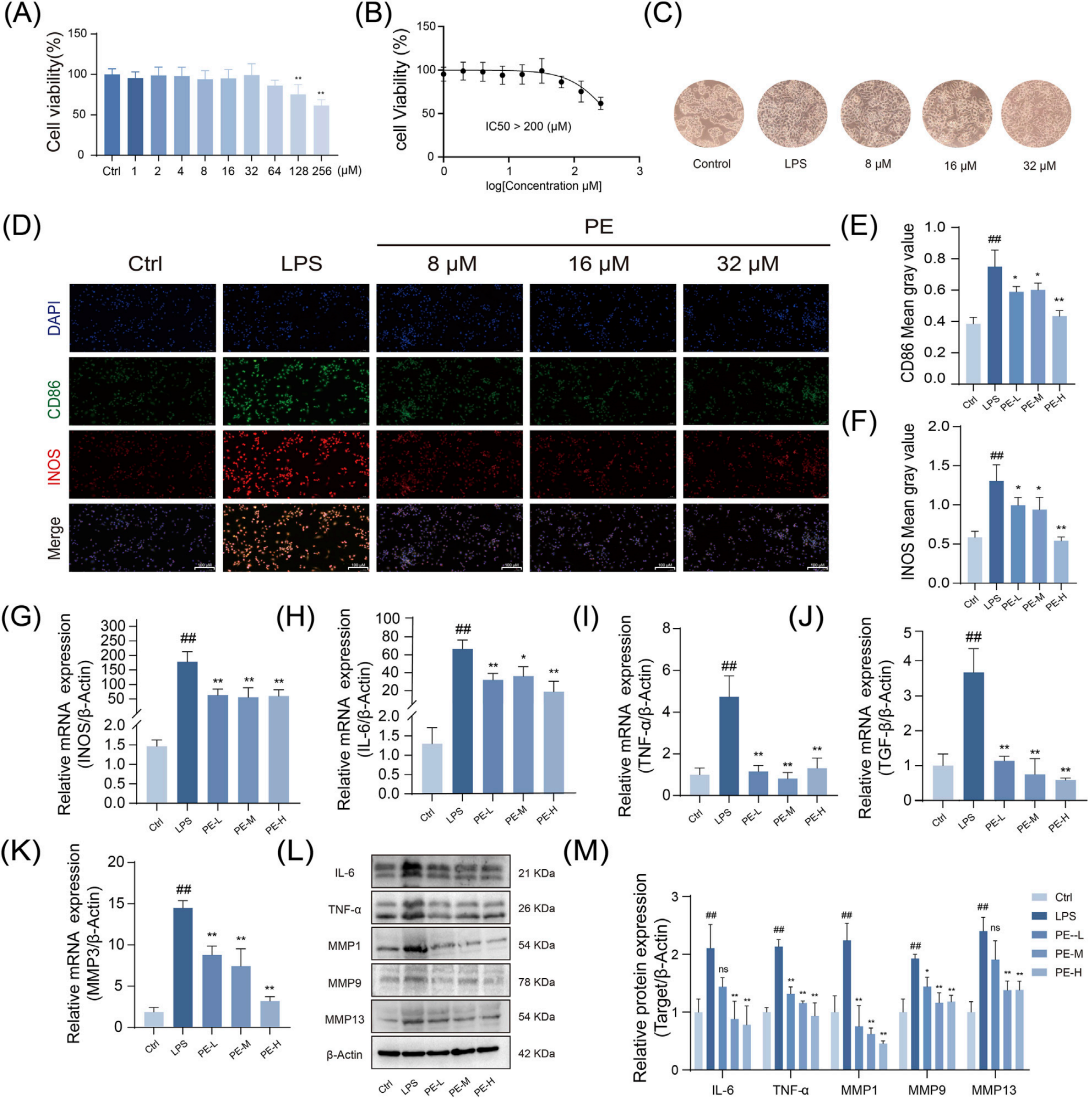
Nti-inflammatory activity of PE against LPS-Induced M1 polarization in RAW264.7 macrophages. **(A)** Assessment of RAW264.7 cell viability after PE treatment; **(B)** Determination of the half-inhibitory concentration of PE on RAW264.7 cells (>200 μM) (n = 6); **(C)** Changes in cell morphology of RAW264.7 cells stimulated with LPS and treated with PE at concentrations of 8, 16, and 32 μM; **(D)** Measurement of fluorescence intensity of CD86 and INOS in RAW264.7 cells via immunofluorescence staining; **(E,F)** Quantification of fluorescence intensity from images **(D)**; **(G–K)** Analysis of gene expression of INOS, IL-6, TNF-α, TGF-β, and MMP3 in RAW264.7 cells after modeling and PE treatment by RT-PCR; **(L)** Evaluation of protein expression of TNF-α, IL-6, MMP1, MMP9, and MMP13 in RAW264.7 cells after modeling and PE treatment; **(M)** Densitometric analysis of the protein expression shown in image **(L)**. Data are the mean ± SD of 3 independent experiments. ^#^ *P* < 0.05, ^##^ *P* < 0.01, vs. Ctrl group; ^*^ *P* < 0.05, ^**^ *P* < 0.01, vs. LPS group; ns, not significant.

Based on previous *in vivo* pharmacodynamic and metabolomic studies which identified PE as a key increased component during processing and the main active constituent entering the bloodstream, this study further validated its direct anti-inflammatory effects through *in vitro* experiments. The results demonstrated that PE significantly inhibits the LPS-induced inflammatory response in macrophages. CCK-8 assay showed that PE had no significant cytotoxicity to RAW264.7 cells at concentrations below 128 μM, and cell viability remained high at 32 μM. Therefore, 8, 16, and 32 μM were selected as the experimental concentrations (Figures 5A,B). LPS treatment significantly induced morphological changes in cells, which could be alleviated by PE (Figure 5C). Immunofluorescence showed PE markedly lowered M1 markers CD86 and INOS, with greater reductions in the high - dose group (Figures 5D–F). RT-PCR and Western blot results both demonstrated that PE significantly inhibited the expression of inflammatory factors such as INOS, IL-6, TNF-α, TGF-β, MMP3, and the proteins MMP1, MMP9, and MMP13 (Figures 5G–M). In summary, PE can effectively alleviate LPS-induced inflammatory responses *in vitro*.

### PE attenuates RA inflammatory responses by modulating the glycolysis pathway

**FIGURE 6:**
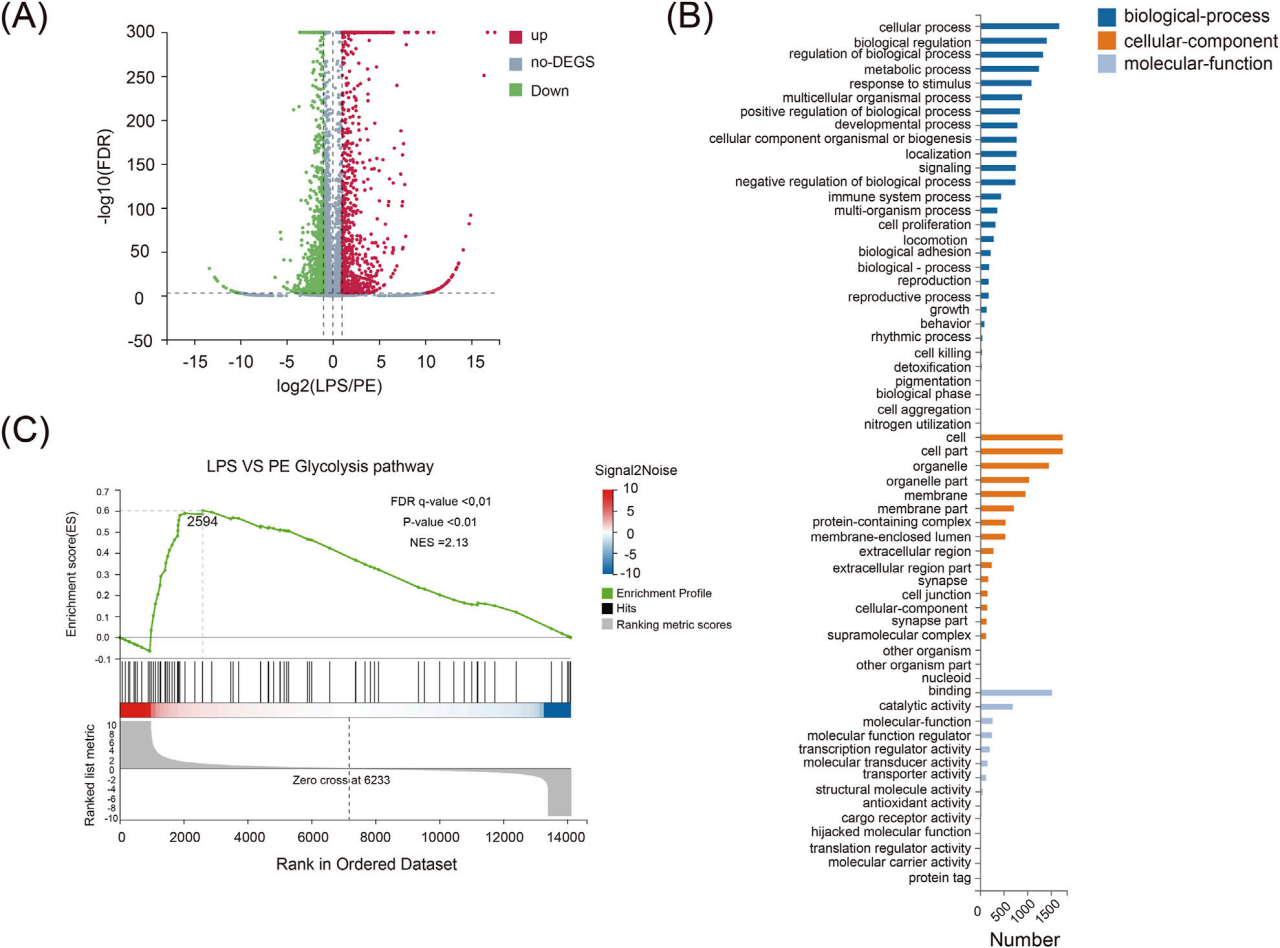
Transcriptome sequencing analysis of differentially expressed genes in RAW264.7 macrophages co-treated with LPS and PE. **(A)** A volcano plot showing differentially expressed genes; **(B)** GO analysis chart; **(C)** GESA analysis chart.

Building on previous findings that demonstrated the significant anti-inflammatory activity of PE *in vitro*, this study further employed integrated transcriptomic and metabolomic analyses to investigate its impact on the glycolytic process, aiming to elucidate the potential mechanisms underlying the alleviation of RA. The results indicate that PE markedly regulates the glycolytic pathway, which is closely associated with its therapeutic effects against RA. LPS-stimulated RAW264.7 cells showed 2095 differentially expressed genes versus controls (908 upregulated, 1187 downregulated) (Figure 6A). GO analysis revealed that these genes were mainly enriched in metabolic processes (Figure 6B), indicating a strong link between RA and glycolytic dysregulation. GSEA analysis demonstrated that LPS significantly activated glycolysis-related pathways, which PE inhibited (Figure 6C). Thus, PE likely alleviates RA by regulating glycolysis-related gene expression, reducing inflammation, and lessening synovial and cartilage damage.

### PE ameliorates metabolic dysregulation in macrophage cells through the inhibition of glycolysis

**FIGURE 7:**
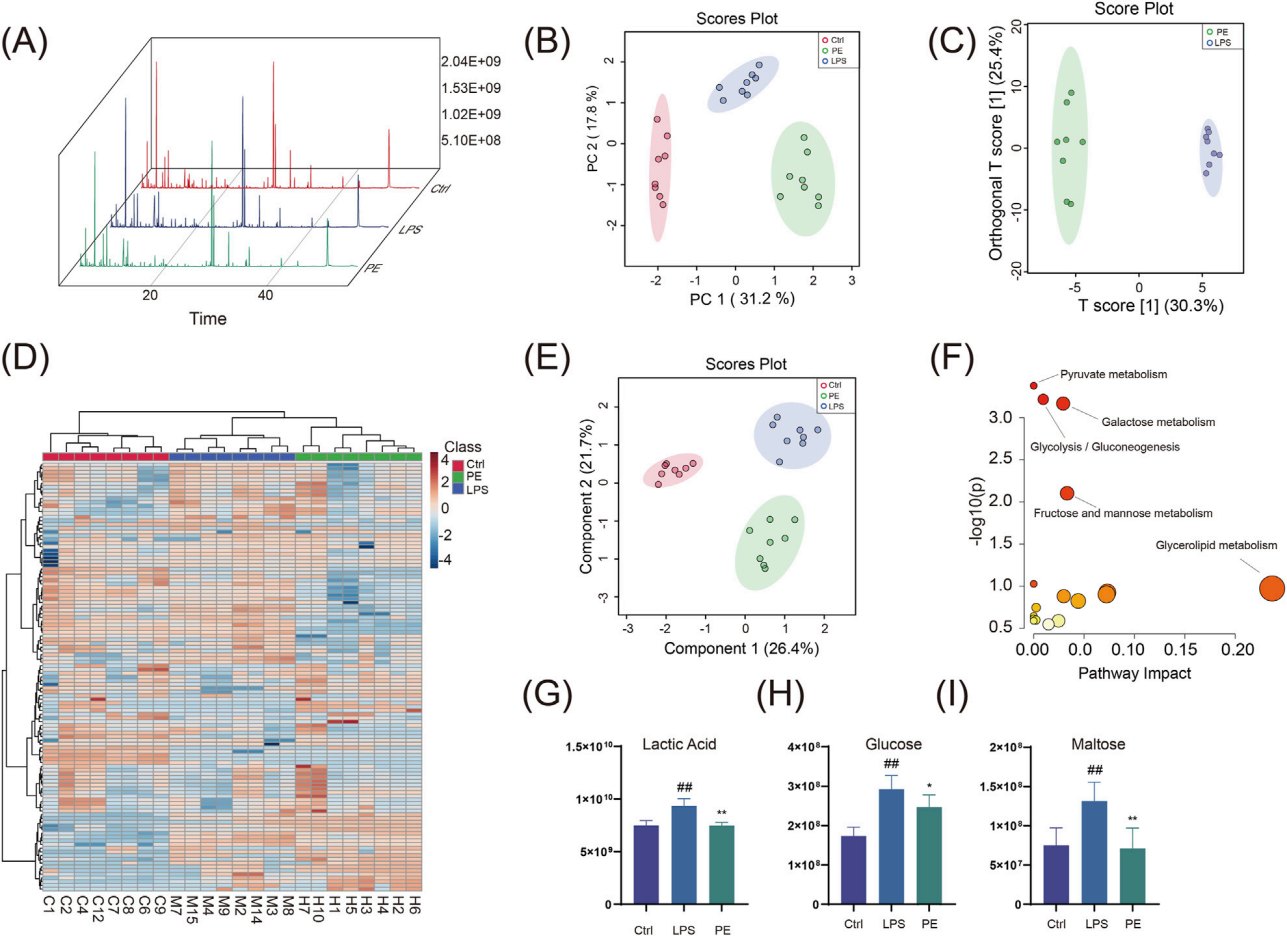
GC-MS Analysis of differential metabolites in RAW264.7 cells co-treated with LPS and PE. **(A)** Total ion current chromatogram; **(B)** PCA plot; **(C)** OPLS-DA plot; **(D)** Heatmap; **(E)** PLS-DA plot; **(F)** Pathway enrichment analysis of differential metabolites; **(G–I)** Peak area statistical charts of differential metabolites. Data are the mean ± SD of 6 independent experiments. ^#^ *P* < 0.05, ^##^ *P* < 0.01, vs. Ctrl group; ^*^ *P* < 0.05, ^**^ *P* < 0.01, vs. LPS group.

Building upon previous evidence that PE modulates glycolytic activity and inflammatory responses, we performed targeted metabolomics to assess how PE alters the metabolic profile of LPS-stimulated RAW264.7 macrophages, aiming to decipher its functional impact on immunometabolic regulation. Our results demonstrate that PE mitigates metabolic dysfunction in macrophages by suppressing glycolytic flux. GC-MS analysis showed that PE treatment significantly changed the metabolite profile of RAW264.7 cells (Figure 7A). PCA, PLS-DA, and OPLS-DA analyses indicated distinct metabolic features among groups (Figures 7B–E). Heatmaps further confirmed significant differences in metabolite profiles (Figure 7D). KEGG analysis showed that PE mainly affected glycolysis/gluconeogenesis and pyruvate metabolism (Figure 7F). Compared with the LPS group, PE significantly decreased lactate, glucose, and maltose levels (Figures 7G–I), suggesting it may reduce lactic acid production and alleviate inflammation by regulating glycolysis.

### PE attenuates cellular inflammation and ameliorates RA *in vitro* model systems by modulating SIRT6-mediated H3K18la and H3K27la

**FIGURE 8:**
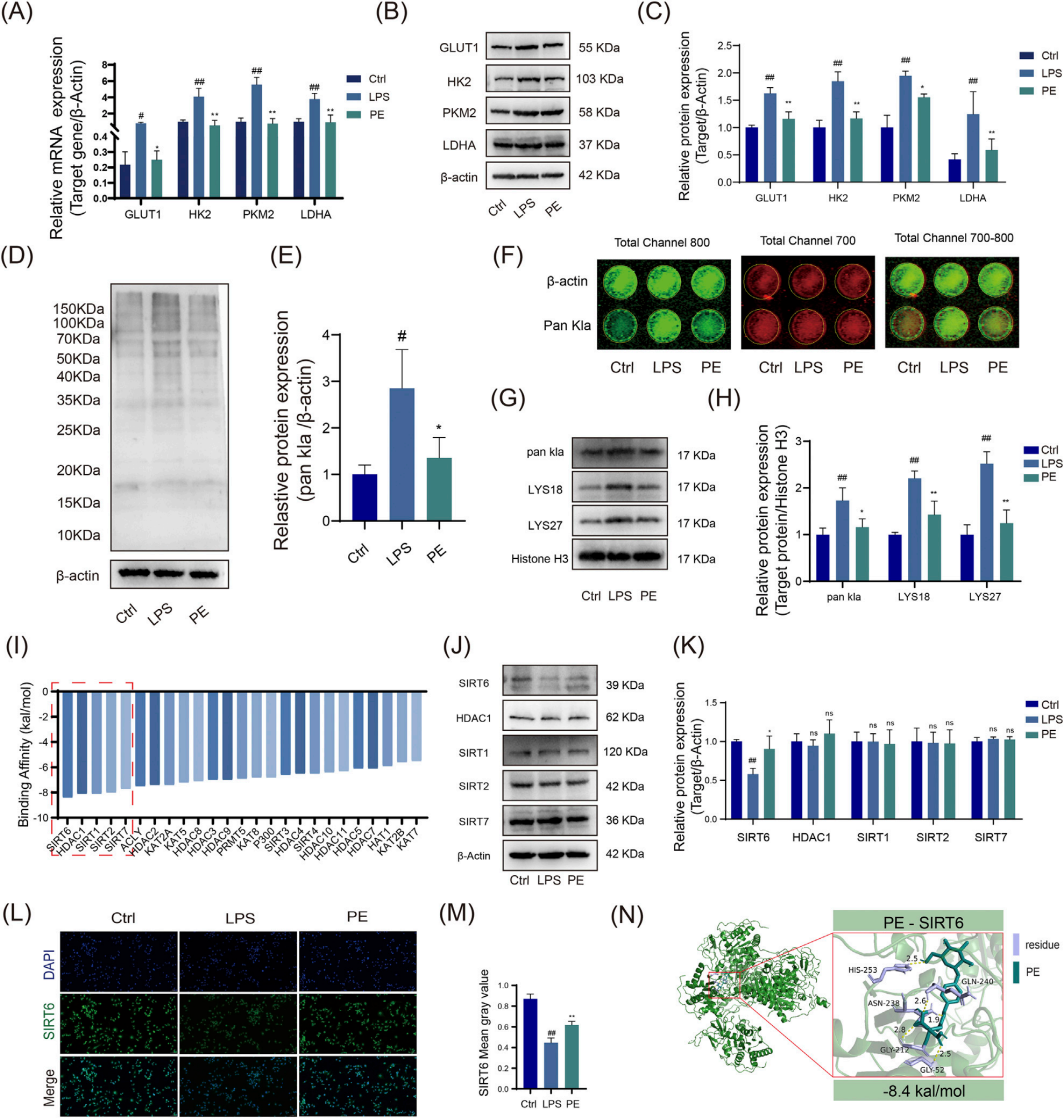
PE modulates M1 macrophage polarization of LPS-induced RAW264.7 cells via the glycolysis pathway *In Vitro*. **(A)** RT - PCR detection of GLUT1, HK2, PKM2, and LDHA at the gene level; **(B)** Western blot analysis of GLUT1, HK2, PKM2, and LDHA protein expression across different treatment groups; **(C)** Quantitative analysis of data from Figure **(B)**; **(D)** Western blot analysis of Pan Kla protein levels in each group; **(E)** Statistical representation of data from Figure **(D)**; **(F)** In - cell WB analysis of Pan Kla expression. **(G,H)** Western blot analysis of nuclear Histone Pan Kla and lysine site modifications in RAW264.7 cells. **(I)** AutoDock Vina molecular docking to predict binding affinity between 25 known acetyltransferases/deacetylases and the small-molecule drug PE. **(J,K)** Western blot analysis of protein expression changes in the top 6 most stably bound enzymes following LPS induction and PE treatment. **(L,M)** Immunofluorescence staining to compare SIRT6 fluorescence intensity across groups. **(N)** Detailed visualization of PE docking with SIRT6. Data are the mean ± SD of 3 independent experiments. ^#^ *P* < 0.05, ^##^ *P* < 0.01, vs. Ctrl group; ^*^ *P* < 0.05, ^**^ *P* < 0.01, vs. LPS group; ns, not significant.

This study preliminarily confirms that PE improves macrophage metabolism and inflammatory phenotypes by regulating glycolysis. To further investigate its epigenetic regulatory mechanisms, we subsequently explored whether PE exerts its effects in RA via SIRT6-mediated histone lactylations (H3K18la/H3K27la). Results indicate that PE significantly attenuates cellular inflammation and ameliorates RA-associated phenotypes *in vitro* by modulating SIRT6-mediated H3K18la and H3K27la lactylations. Glycolysis, regulated by key enzymes like GLUT1, HK2, PKM2, and LDHA, was enhanced in LPS-stimulated RAW264.7 cells via significant upregulation of these enzymes. However, PE treatment markedly reduced their mRNA and protein levels, thereby inhibiting glycolysis (Figures 8A–C). In addition, PE effectively decreased global protein lactylation levels, modulating this post-translational modification (Figures 8D,E). The In-cell Western blot results matched those of the Western blot (Figure 8F).

LPS stimulation increased H3K18la and H3K27la lactylation in RAW264.7 cells, while PE treatment inhibited this modification (Figures 8G,H). Molecular docking predictions showed that PE had the strongest binding energy with SIRT6 (−8.4 kcal/mol), suggesting it may be a primary target (Figure 8I). Western blotting showed that PE reversed LPS-induced SIRT6 downregulation, suggesting PE modulates RA via SIRT6-mediated H3K18la and H3K27la changes (Figures 8J,K).

**FIGURE 9:**
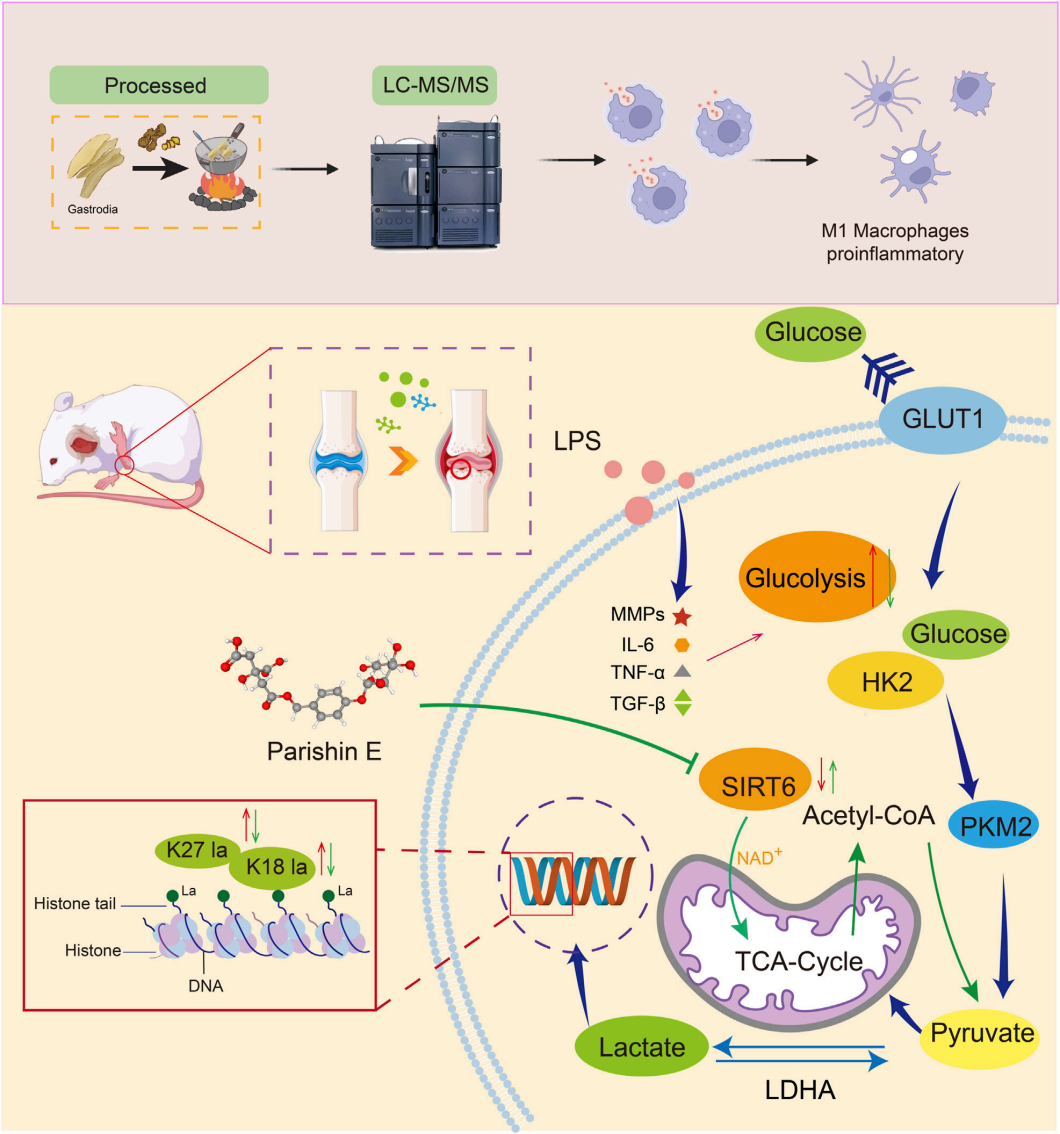
Overall mechanism diagram of the experiment.

Immunofluorescence results showed that PE increased SIRT6 expression, consistent with Western blot results (Figures 8L,M). Molecular docking diagrams revealed stable hydrogen bonds and key amino acid residues between PE and SIRT6 (Figure 8N), indicating that PE can stably bind to SIRT6 in cells and regulate its function. In summary, PE significantly inhibits inflammatory responses and improves the pathological state of RA by regulating SIRT6-mediated H3K18la and H3K27la lactylation modifications (Figure 9).

## Discussion

Processing GEB with ginger juice yields a more moderate product. The active components in ginger juice, including gingerols, alkaloids, and volatile oils, enhance the dissolution and absorption of natural bioactive constituents in G-GEB. This processing method increases the bioavailability of compounds such as PE and potentiates their pharmacological effects (Ye et al., 2019). GEB alleviates rheumatic arthralgia and peripheral neuropathic numbness through its anti-inflammatory and neurotrophic effects, while ginger juice synergistically enhances therapeutic efficacy via vasodilation, thermogenic regulation, anti-edema, and analgesic mechanisms (Tang et al., 2015). The combination of G-GEB and ginger juice exerts synergistic therapeutic effects on rheumatoid disorders (wind-cold-dampness arthralgia syndrome) through multi-target mechanisms. However, limited research has been conducted on the material basis for the enhanced efficacy during processing. Integrating non-targeted plant metabolomics can help elucidate the transformation mechanisms of active ingredients during the processing (Castro-Alves et al., 2021; Ge et al., 2023).

Combined plant non-targeted metabolomics and plasma metabolomics analysis revealed that PE is a component that significantly increases and is detected in the bloodstream after ginger juice processing. It may be derived from the enzymatic hydrolysis of its precursors, including Parishin A, B, C, and glycosides (Zhang et al., 2024). The elevation of cordycepin content may be due to the enzymatic transformation of adenosine catalyzed by enzymes in ginger juice (Turk et al., 2023); Citric acid, derived from ginger juice, remains stable under acidic conditions and its content increases during the processing (Liu et al., 2023). The primary active component in ginger juice, 6-Gingerol, may be incorporated into the Gastrodia elata tissue during the heating process, thereby increasing the measurable concentration of 6-Gingerol (Zhang et al., 2021). Additionally, its derivative [6]-Gingerdiol 3.5-diacetate may be generated by oxidation or esterification, and the increase in its content may be associated with enzymatic reactions in ginger juice or heat-induced transformation of ginger juice components.

The identification of blood components revealed that PE was the most abundant substance among the absorbed components, indicating its favorable systemic absorption and exposure, and suggesting its potential as a key material basis for pharmacological effects. Integrated with non-targeted metabolomics results, comparison of peak areas between GEB and G-GEB showed a significant increase in PE content after processing, leading to the hypothesis that PE may be a critical differential component responsible for the efficacy enhancement of GEB following ginger juice processing. Preliminary results demonstrated that PE exhibits significant and stable anti-inflammatory activity *in vitro*. Furthermore, existing literature has reported that PE is an important active component in GEB with potent anti-inflammatory properties. Therefore, PE was selected as a candidate molecule for further in-depth investigation into its mechanism for treating rheumatoid arthritis.

Despite multiple studies demonstrating that the content of PE in Gastrodia elata first increases and then decreases after being processed with various adjuvants (Zhang et al., 2024; Li et al., 2019; Cheng et al., 2023), no reports have been published yet on the effect of PE in alleviating diseases. The *in vitro* RA model induced by LPS-stimulated macrophages is a common strategy in current studies on inflammatory diseases, and it is particularly suitable for investigating the pathogenesis of RA, inflammatory signaling pathways, and screening for potential anti-inflammatory candidates (Ma et al., 2022; Zeng et al., 2016). PE significantly inhibits inflammatory responses. To further elucidate the molecular mechanisms underlying the anti-inflammatory effects of PE in an *in vitro* model of RA, we performed transcriptomic and metabolomic analyses of PE-treated inflammatory macrophages. These multi-omics analyses revealed that the anti-inflammatory effects of PE are closely associated with its systemic regulation of glycolytic metabolism.

Previous studies have shown that glycolysis plays a crucial role in RA. LPS stimulation of macrophages can increase lactate levels, which in turn regulates pathological processes via lysine lactylation (Kla). PE can regulate Kla expression at histone H3K18/27 sites via SIRT6-mediated deacetylation. Despite the mechanisms revealed in this study, there are limitations: First, the non-targeted metabolomics samples were all from Hubei, and the anti-inflammatory effects of PE need to be verified with a broader sample source. Second, the anti-inflammatory mechanism of PE is limited to *in vitro* studies, and its *in vivo* anti-RA efficacy and mechanisms need further investigation.

This study demonstrates that processing with ginger juice can significantly enhance the anti-RA effects of Gastrodia elata. Compared with GEB, G-GEB is more effective in alleviating joint swelling, improving cartilage structure, and regulating immune function. Non-targeted metabolomics and pharmacokinetics analyses indicate that G-GEB is rich in various active components, such as Parishin E and [6]-gingerol. *In vitro* studies further confirmed that Parishin E can effectively inhibit M1 polarization of macrophages by suppressing the expression of pro-inflammatory cytokines and regulating glycolysis. In summary, ginger juice processing enhances the anti-inflammatory mechanisms of GEB, providing new insights and potential drug candidates for the treatment of RA.

## Conclusion

Our study shows G-GEB improves the anti-RA efficacy of GEB. Metabolomics analysis finds PE as a key active component in G-GEB. PE has anti-inflammatory effects, which may be related to glycolysis regulation and M1 macrophage polarization inhibition. It also affects histone lactylation via SIRT6. Despite limitations, our work backs the clinical use of G-GEB and suggests PE as a potential RA therapy. Future research should further test PE’s *in vivo* effects.

## Data Availability

The dataset presented in this study can be found in the NCBI (BioProject) repository, accession number PRJNA1347736. Available at https://www.ncbi.nlm.nih.gov/bioproject/PRJNA1347736.

## Glossary

GEB: Gastrodia elata Bl.
G-GEB: Ginger - processed Gastrodia elata Bl.
RA: Rheumatoid Arthritis
SD: Sprague Dawley
CFA: Complete Freund’s adjuvant
LC-MS/MS: Liquid Chromatography-mass spectrometry
GC-MS: Gas Chromatography-Mass Spectrometry
AIA: Antigen-Induced Arthritis
LPS: Lipopolysaccharides
PE: Parishin E
INOS: Inducible Nitric Oxide Synthase
TNF-α: Tumor Necrosis Factor-alpha
IL-6: Interleukin-6
TGF-β: Transforming Growth Factor-beta
MMP1: Matrix Metalloproteinase-1
MMP9: Matrix Metalloproteinase-9
MMP13: Matrix Metalloproteinase-13
MMPs: Matrix Metalloproteinases
GLUT1: Glucose Transporter 1
HK2: Hexokinase 2
PKM2: Pyruvate Kinase M2
LDHA: Lactate Dehydrogenase A
SIRT6: Sirtuin 6
HDAC1: Histone Deacetylase 1
SIRT1: Sirtuin 1
SIRT2: Sirtuin 2
SIRT7: Sirtuin 7
MTX: Methotrexate
AI: Arthritis Scoring Artificial Intelligence
EDTA: Ethylene Diamine Tetraacetic Acid
H&E: Hematoxylin and Eosin
OARSI: Osteoarthritis Research Society International
HESI: Heated Electrospray Ionization
DDA: Data Dependent Acquisition
PDA: Photo-Diode Array
FBS: Fetal Bovine Serum
DMEM: Dulbecco’s Modified Eagle Medium
CCK-8: Cell Counting Kit-8
BCA: Bicinchoninic Acid Assay
SDS-PAGE: Sodium Dodecyl Sulfate-Polyacrylamide Gel Electrophoresis
PVDF: Polyvinylidene fluoride
RT-PCR: Reverse Transcription-Polymerase Chain Reaction
PBST: Phosphate-Buffered Saline with Tween-20
CD86: Cluster of Differentiation 86
DAPI: 4′,6-Diamidino-2-Phenylindole
GESA: Gene Set Enrichment Analysis
BSTFA: N,O-Bis(trimethylsilyl)trifluoroacetamide
DEGs: Differentially Expressed Genes
GO: Gene Ontology Enrichment Analysis.
