# Supplementary material for: Parishin E from ginger-processed Gastrodia elata Bl. alleviates rheumatoid arthritis by regulating histone 3 lactylation at H3K18la and H3K27la sites
Source: Front Pharmacol. 2025 Oct 28;16:1682504. doi: 10.3389/fphar.2025.1682504 (PMC12602504; doi:10.3389/fphar.2025.1682504)
Supplement: Supplementary file 1 [file DataSheet1.zip › Table Supplementary/Table Supplementary 1.docx]

| **Table S1: GEB processed with ginger juice exhibits elevated levels of its top 50 chemical constituents** | | | | | | | | | | | | | | |
| --- | --- | --- | --- | --- | --- | --- | --- | --- | --- | --- | --- | --- | --- | --- |
| **ID** | **m/z** | **Retention time (min)** | **Ion mode** | **Metabolites** | **Formula** | **Fragmentation Score** | **Fragment Ions** | **HMDB** | **CAS** | **VIP** | **log2FoldChange** | **Fold Change** | **p-value** | **q-value** |
| 1 | 403.2085 | 10.9541 | pos | [6]-Gingerdiol 3,5-diacetate | C21H32O6 | 90.7 | 129.0181, 137.0596, 157.0128, 163.0752, 185.0806, 208.8278, 210.0443, 216.0304, 343.1845, 403.2078 | HMDB0040568 | 143615-75-2 | 4.2965 | 12.4815 | 5719.0859 | < 0.0001 | 0.0009 |
| 2 | 373.1654 | 6.7221 | neg | Hexahydrocurcumin | C21H26O6 | 57.8 | 57.0344, 121.0293, 164.0473, 165.0554, 174.9557, 179.0710, 193.0867, 304.9144, 372.8655, 373.1649 | HMDB0302135 |  | 3.7131 | 9.3341 | 645.4425 | < 0.0001 | 0.0016 |
| 3 | 305.2105 | 10.3676 | pos | ent-15,16-epoxy-3-oxa-kauran-2-one | C19H28O3 | 0 | 69.0704, 117.0704, 137.0596, 145.0646, 162.0675, 169.1585, 177.0907, 179.0698, 287.2002, 305.1569 | N/A |  | 3.6749 | 9.227 | 599.2657 | 0.0002 | 0.0097 |
| 4 | 483.198 | 9.444 | pos | paraaspidin bb | C25H32O8 | 0 | 107.0491, 137.0594, 163.0743, 208.1760, 215.0157, 363.1577, 423.1691, 423.1759, 483.1113, 483.1974 | N/A |  | 3.6961 | 9.2228 | 597.5379 | < 0.0001 | <0.0001 |
| 5 | 432.2486 | 5.1059 | pos | desacetylvindoline | C23H30N2O5 | 0 | 90.0553, 98.9846, 107.0494, 110.0714, 137.0596, 156.0766, 271.0598, 365.1042, 386.2424, 432.2488 | N/A |  | 3.5377 | 8.4968 | 361.2594 | < 0.0001 | 0.0047 |
| 6 | 375.1812 | 6.2481 | neg | (3R,5R)-1,7-bis(4-hydroxy-3-methoxyphenyl)heptane-3,5-diol | C21H28O6 | 0 | 137.0597, 163.0753, 177.0903, 183.6792, 208.4211, 217.1227, 271.0653, 341.1750, 377.0644, 377.0710 | N/A | 36062-07-4 | 3.4771 | 8.1843 | 290.9013 | < 0.0001 | 0.0016 |
| 7 | 405.1914 | 6.1027 | neg | Nigakilactone K | C22H30O7 | 0 | 73.9892, 123.7645, 131.0749, 148.3512, 207.9263, 214.6737, 219.1814, 296.3838, 405.1903, 451.1318 | N/A |  | 3.4545 | 8.0764 | 269.9277 | < 0.0001 | 0.0016 |
| 8 | 361.1976 | 9.898 | pos | Shiromodiol diacetate | C19H30O5 | 0 | 73.0289, 131.0490, 137.0595, 163.0752, 177.0902, 209.0238, 212.0747, 261.1841, 279.1950, 356.0696 | N/A | 20071-58-3 | 3.3820 | 7.7577 | 216.4246 | < 0.0001 | 0.0037 |
| 9 | 389.1599 | 5.6695 | neg | (-)-5'-Methoxyisolariciresinol | C21H26O7 | 0 | No MS/MS spectrum detected | N/A |  | 3.2374 | 7.0781 | 135.1275 | < 0.0001 | 0.0000 |
| 10 | 261.1845 | 9.9193 | pos | Panaxacol | C17H26O3 | 0 | 131.0489, 137.0600, 145.0643, 159.0804, 163.0754, 177.0907, 191.1068, 229.1582, 261.1308, 261.1833 | HMDB0039251 | 106828-96-0 | 3.2095 | 7.0017 | 128.1568 | < 0.0001 | 0.0059 |
| 11 | 179.0700 | 9.0736 | pos | Xanthoxylin | C10H12O4 | 9.94 | 56.9653, 108.0446, 111.9682, 116.9715, 121.0648, 134.0603, 137.0598, 138.0633, 152.0703, 165.0912 | HMDB0029645 | 90-24-4 | 3.1934 | 6.9347 | 122.3358 | 0.0001 | 0.0059 |
| 12 | 261.1846 | 10.9541 | pos | 1-(4-Hydroxy-3-methoxyphenyl)-3-decanone | C17H26O3 | 48.9 | 131.0488, 137.0596, 145.0643, 159.0803, 163.0751, 177.0908, 191.1062, 229.1582, 261.1329, 261.1840 | HMDB0030801 | 27113-22-0 | 3.1669 | 6.8212 | 113.0804 | 0.0001 | 0.0059 |
| 13 | 277.1793 | 9.0736 | pos | 6-Gingerol | C17H26O4 | 92.3 | 83.0859, 117.0699, 137.0595, 141.1270, 145.0645, 162.0672, 163.0750, 177.0906, 259.1685, 277.1790 | N/A | 23513-14-6 | 3.1571 | 6.7808 | 109.9579 | 0.0001 | 0.0059 |
| 14 | 371.1844 | 6.0833 | pos | amethystonal | C20H28O5 | 0 | 129.0545, 137.0599, 147.0655, 163.0755, 167.0703, 193.0859, 207.1014, 217.1228, 355.0692, 371.1852 | N/A |  | 3.1475 | 6.7081 | 104.5543 | < 0.0001 | 0.0017 |
| 15 | 275.2001 | 10.5121 | pos | polyacetylene PQ-1 | C18H28O3 | 0.171 | 81.0703, 151.0753, 160.0877, 165.0910, 177.0908, 189.0909, 191.1064, 205.1220, 221.1537, 275.2001 | N/A |  | 3.1113 | 6.6973 | 103.7781 | 0.0009 | 0.0233 |
| 16 | 401.1949 | 9.4440 | pos | Gomisin S | C23H30O7 | 0 | 131.0490, 137.0597, 163.0753, 177.0908, 185.0970, 208.7801, 209.0341, 209.0910, 217.1224, 341.1714 | N/A |  | 3.1220 | 6.5816 | 95.7817 | < 0.0001 | 0.0000 |
| 17 | 429.1875 | 6.104 | pos | 1-(4-Hydroxy-3,5-dimethoxyphenyl)-7-(4-hydroxy-3-methoxyphenyl)-3,5-heptanediol | C22H30O7 | 55.1 | 98.9842, 136.0616, 208.3788, 208.4036, 208.9534, 318.9218, 359.0270, 429.0823, 429.1092, 429.1876 | HMDB0041091 | 145888-84-2 | 3.1036 | 6.5479 | 93.5714 | < 0.0001 | 0.0059 |
| 18 | 294.2056 | 5.5431 | pos | nobilonine | C17H27NO3 | 0 | 100.1124, 107.0489, 131.0493, 137.0593, 210.5993, 211.0711, 259.0925, 277.1017, 294.1745, 294.2051 | N/A |  | 3.0922 | 6.4641 | 88.2887 | < 0.0001 | 0.0009 |
| 19 | 333.2419 | 12.4195 | pos | [10]-Shogaol | C21H32O3 | 39.2 | 89.0599, 107.9671, 137.0596, 137.0727, 151.0960, 184.8883, 333.1503, 333.2016, 333.2307, 333.2421 | HMDB0031462 | 36752-54-2 | 3.0542 | 6.3668 | 82.5292 | 0.0001 | 0.0080 |
| 20 | 345.2031 | 10.3676 | pos | (8)-Gingerol | C19H30O4 | 40.9 | 64.1143, 67.0495, 74.4475, 78.0159, 104.3168, 128.2859, 214.7217, 251.5443, 345.1560, 345.2031 | N/A | 23513-08-8 | 2.9980 | 6.1459 | 70.8117 | 0.0002 | 0.0100 |
| 21 | 445.1866 | 8.4048 | neg | ethoxyclusin | C23H28O8 | 0 | 59.0138, 102.9570, 208.9493, 210.7313, 215.9934, 302.8903, 305.1531, 325.1420, 385.1643, 445.1864 | N/A |  | 2.9597 | 6.1195 | 69.5280 | 0.0015 | 0.0343 |
| 22 | 427.1717 | 6.5582 | pos | caesalpinin o | C22H28O7 | 0 | 137.0598, 163.0760, 167.0702, 175.0747, 177.0918, 193.0862, 207.1013, 208.0442, 242.3536, 369.1712 | N/A |  | 2.9589 | 5.9966 | 63.8495 | 0.0003 | 0.0108 |
| 23 | 431.1707 | 7.2569 | neg | norcaesalpinin md | C23H28O8 | 0 | 88.9879, 158.9785, 189.0916, 208.8910, 226.9666, 294.9570, 311.1300, 371.1499, 412.8249, 431.1711 | N/A |  | 2.8615 | 5.9185 | 60.4851 | 0.0059 | 0.0760 |
| 24 | 531.1467 | 5.0650 | pos | specioside | C24H28O12 | 0 | 122.0373, 122.6131, 180.7843, 181.4184, 213.5039, 257.8592, 277.0680, 277.7530, 509.0357, 531.1470 | N/A |  | 2.7972 | 5.8541 | 57.8455 | 0.0130 | 0.1144 |
| 25 | 321.2054 | 10.9541 | pos | [(1S,5S,7S)-7-acetoxy-5-isopropenyl-2,8-dimethylene-cyclodecyl] acetate | C19H28O4 | 0 | 81.0700, 131.0488, 137.0595, 145.0650, 163.0751, 177.0908, 191.1061, 229.1576, 261.1841, 321.2028 | N/A |  | 2.9163 | 5.7954 | 55.5401 | 0.0001 | 0.0075 |
| 26 | 177.0908 | 9.0736 | pos | Butylparaben | C11H14O3 | 0 | 91.0546, 95.0859, 107.0854, 115.0536, 117.0700, 121.0646, 121.1012, 145.0645, 149.0229, 177.0906 | HMDB0032575 | 94-26-8 | 2.9019 | 5.7464 | 53.6834 | 0.0002 | 0.0086 |
| 27 | 291.1582 | 11.0589 | pos | [6]-Dehydrogingerdione | C17H22O4 | 84.8 | 71.0861, 99.0808, 141.0911, 145.0285, 151.0753, 177.0544, 177.0910, 241.1214, 273.1484, 291.1585 | HMDB0029474 | 76060-35-0 | 2.8893 | 5.7076 | 52.2614 | 0.0002 | 0.0097 |
| 28 | 259.1688 | 9.0736 | pos | Sugeonyl acetate | C17H24O3 | 0.729 | 137.0595, 143.0488, 163.0752, 171.0798, 175.0751, 185.0960, 189.0907, 203.1064, 227.1429, 259.1688 | N/A |  | 2.8706 | 5.6313 | 49.5689 | 0.0002 | 0.0097 |
| 29 | 441.1874 | 7.9934 | pos | Diosbulbin H | C23H30O7 | 0 | 56.8230, 70.3053, 75.7974, 94.6762, 207.9709, 441.1879 | HMDB0036781 | 67567-13-9 | 2.8590 | 5.5204 | 45.8995 | < 0.0001 | 0.0001 |
| 30 | 300.1798 | 4.3898 | pos | Suberoyl-L-carnitine | C15H27NO6 | 0 | No MS/MS spectrum detected | HMDB0240724 |  | 2.7960 | 5.4759 | 44.5065 | 0.0019 | 0.0373 |
| 31 | 431.1708 | 7.8949 | neg | (8r,8'r,9's)-5-methoxyclusin | C23H28O8 | 0 | No MS/MS spectrum detected | N/A |  | 2.8434 | 5.4628 | 44.1039 | < 0.0001 | 0.0005 |
| 32 | 395.1456 | 6.9739 | pos | (-)-Phillygenin | C21H24O6 | 0 | 50.9570, 52.1620, 84.6386, 91.0719, 207.9807, 208.0042, 296.1695, 307.4877, 314.0173, 395.1460 | N/A | 487-39-8 | 2.8202 | 5.4181 | 42.7579 | 0.0001 | 0.0075 |
| 33 | 153.1021 | 4.0326 | pos | (3xi,6xi)-Cyclo(alanylvalyl) | C8H14N2O2 | 0 | No MS/MS spectrum detected | N/A |  | 2.7479 | 5.3950 | 42.0792 | 0.0046 | 0.0619 |
| 34 | 350.2320 | 5.6244 | pos | (5S,6S,15R)-15-Methyllycopodane-5,6-diol 5,6-diacetate | C20H31NO4 | 0 | 107.0493, 137.0596, 154.1595, 156.1383, 296.0072, 324.0064, 332.2222, 349.9821, 350.1135, 350.2331 | N/A |  | 2.7744 | 5.1981 | 36.7105 | < 0.0001 | 0.0000 |
| 35 | 390.2265 | 6.2088 | pos | tuberostemonol | C22H31NO5 | 0 | 51.1663, 138.0633, 149.0230, 168.0729, 180.1017, 181.1098, 211.3872, 250.1796, 266.1027, 390.2275 | N/A |  | 2.7351 | 5.1572 | 35.6843 | 0.0007 | 0.0187 |
| 36 | 377.1948 | 6.2894 | pos | 1,7-bis-(4-Hydroxy-3-methoxyphenyl)-heptane-3,5-diol | C21H28O6 | 58.7 | 137.0597, 163.0753, 177.0903, 183.6792, 208.4211, 217.1227, 271.0653, 341.1750, 377.0644, 377.0710 | HMDB0303086 |  | 2.6989 | 4.9749 | 31.4501 | 0.0002 | 0.0094 |
| 37 | 213.1231 | 3.8983 | pos | N-acetyldopamine | C10H13NO3 | 19.5 | 91.0546, 119.0492, 135.0916, 137.0596, 149.1074, 150.0184, 177.1022, 195.1127, 213.0902, 213.1220 | HMDB0041943 | 2494-12-4 | 2.6875 | 4.9742 | 31.4338 | 0.0005 | 0.0148 |
| 38 | 201.1022 | 5.4606 | pos | cyclo-(phe-ala) | C12H14N2O2 | 0 | No MS/MS spectrum detected | N/A |  | 2.6774 | 4.9098 | 30.0625 | 0.0002 | 0.0099 |
| 39 | 291.1600 | 9.0730 | neg | pathenolide | C16H22O2 | 0 | No MS/MS spectrum detected | N/A |  | 2.6659 | 4.8754 | 29.3540 | 0.0004 | 0.0134 |
| 40 | 289.1445 | 9.5826 | neg | kushequinone A | C17H22O4 | 0 | No MS/MS spectrum detected | N/A | 102390-90-9 | 2.6612 | 4.8677 | 29.1974 | 0.0005 | 0.0154 |
| 41 | 518.2312 | 6.4686 | pos | Lythramine | C29H37NO5 | 0 | 149.0608, 149.5617, 175.0396, 176.0481, 191.0714, 206.3397, 207.8334, 209.7292, 242.6853, 291.1603 | N/A | 32420-56-7 | 2.6141 | 4.8403 | 28.6467 | 0.0034 | 0.0550 |
| 42 | 317.2470 | 11.1009 | pos | 5alpha-Pregnane-3,20-dione | C21H32O2 | 0 | No MS/MS spectrum detected | HMDB0003759 | 566-65-4 | 2.6130 | 4.7914 | 27.6929 | 0.0023 | 0.0415 |
| 43 | 224.1025 | 1.1735 | pos | 5-Methyldeoxycytidine | C10H15N3O4 | 0 | No MS/MS spectrum detected | HMDB0002224 | 838-07-3 | 2.5703 | 4.7868 | 27.6044 | 0.0066 | 0.0773 |
| 44 | 391.1760 | 5.5249 | neg | hancopregnane | C21H28O7 | 62.6 | 135.0454, 151.0399, 186.9385, 214.6822, 236.9106, 248.5292, 345.0100, 345.1180, 376.1515, 391.1764 | N/A |  | 2.5845 | 4.7666 | 27.2213 | 0.0039 | 0.0592 |
| 45 | 139.0866 | 4.1489 | pos | Benzamide | C7H7NO | 0 | No MS/MS spectrum detected | HMDB0004461 | 55-21-0 | 2.6095 | 4.6699 | 25.4568 | 0.0003 | 0.0120 |
| 46 | 235.1077 | 4.7919 | pos | 4-hydroxy mephenytion | C12H14N2O3 | 0 | No MS/MS spectrum detected | N/A | 61837-65-8 | 2.5946 | 4.6484 | 25.0796 | 0.0006 | 0.0177 |
| 47 | 355.1549 | 8.5573 | neg | (1R,5R,6R,7R)-3-allyl-6-(3,4-dimethoxyphenyl)-1-methoxy-7-methylbicyclo[3.2.1]oct-2-ene-4,8-dione | C21H24O5 | 0 | No MS/MS spectrum detected | N/A |  | 2.6090 | 4.6044 | 24.3260 | < 0.0001 | 0.0012 |
| 48 | 371.1845 | 9.2586 | pos | glyasperins D | C22H26O5 | 0 | No MS/MS spectrum detected | N/A | 142561-10-2 | 2.5819 | 4.5233 | 22.9966 | < 0.0001 | 0.0040 |
| 49 | 343.1547 | 6.5399 | neg | ailantinol f | C20H26O6 | 0 | No MS/MS spectrum detected | N/A |  | 2.5738 | 4.5220 | 22.9756 | 0.0002 | 0.0088 |
| 50 | 543.2069 | 4.9813 | neg | taxagifine iii | C24H34O11 | 0 | No MS/MS spectrum detected | N/A |  | 2.5521 | 4.4847 | 22.3895 | 0.0006 | 0.0174 |
