# Supplementary material for: Parishin E from ginger-processed Gastrodia elata Bl. alleviates rheumatoid arthritis by regulating histone 3 lactylation at H3K18la and H3K27la sites
Source: Front Pharmacol. 2025 Oct 28;16:1682504. doi: 10.3389/fphar.2025.1682504 (PMC12602504; doi:10.3389/fphar.2025.1682504)
Supplement: Supplementary file 1 [file DataSheet1.zip › Table Supplementary/Table Supplementary 2.docx]

| **Table S2: Identification of 11 Blood-Borne Components** | | | | | | | | | | | | | | |
| --- | --- | --- | --- | --- | --- | --- | --- | --- | --- | --- | --- | --- | --- | --- |
| **No** | **Metabolites** | **Formula** | **Fragmentation Score** | **Fragment Ions** | **theoretical m/z** | **Retention time (min)** | **Ion mode** | **C Mean** | **g-GEB Mean** | **TCM Mean** | **Ratio of Peak Area %** | **CAS** | **English Category** | **HMDB** |
| 1 | Gastrodin | C13H18O7 | 68.3 | 123.0449, 161.0439, 285.0991, 331.1003, 331.1056 | 331.1035 | 1.86 | NEG | 0 | 18647.50248 | 29349933.41 | 3.982957029 | 62499-27-8 | Carbohydrates and Glycosides | N/A |
| 2 | Parishin E | C19H24O13 | 50.4 | 107.0494, 215.0155, 377.066, 483.1103 | 483.1110 | 3.96 | POS | 0 | 42387.74556 | 59598009.94 | 8.087797314 | 952068-57-4 | Alkaloids | N/A |
| 3 | Ergothioneine | C9H15N3O2S | 98.7 | 60.0814, 127.0323, 186.1054, 230.0944 | 230.0958 | 1.08 | POS | 0 | 3181.912496 | 6983922.901 | 0.947759043 | [497-30-3](https://www.chemsrc.com/baike/33351.html) | Alkaloids | HMDB0003045 |
| 4 | Pelargonic acid | C9H18O2 | 66.4 | 314.9826, 315.2536 | 315.2541 | 10.49 | NEG | 12805.72214 | 40995.19451 | 2720.436395 | 0.000369179 | [112-05-0](https://www.chemsrc.com/baike/335862.html) | Fatty Acyls | HMDB0000847 |
| 5 | 4-Hydroxyisoleucine | C6H13NO3 | 0 | No MS/MS spectrum detected | 148.0968 | 1.08 | POS | 4957.836481 | 10078.78345 | 660108.1102 | 0.089580518 | 55399-93-4 | Amino Acids, Peptides and derivatives | N/A |
| 6 | Epoxyoleic acid | C18H34O3 | 0 | No MS/MS spectrum detected | 299.2581 | 11.70 | POS | 6662.386995 | 19158.61956 | 4461.176671 | 0.000605408 | 24560-98-3 | Others | N/A |
| 7 | [10]-Shogaol | C21H32O3 | 36 | 137.0595, 333.1516, 333.2025 | 333.2424 | 12.26 | POS | 0 | 2069.041429 | 28720.74436 | 0.003897572 | 36752-54-2 | Phenols | HMDB0031462 |
| 8 | Griffonilide | C8H8O4 | 14.9 | 129.0193, 145.0508, 146.0454, 151.0008, 151.0400, 169.0137, 169.0501, 183.0296, 213.0025, 213.0397 | 213.0405 | 2.00 | NEG | 0 | 2239.419983 | 32624.18088 | 0.004427291 | 61371-55-9 | Others | N/A |
| 9 | N2-Methylguanosine | C11H15N5O5 | 41.2 | 107.0493, 136.0617, 152.0564, 166.0721, 298.0865 | 298.1146 | 2.06 | POS | 0 | 1823.049549 | 83800.18227 | 0.011372173 | 2140-77-4 | Others | HMDB0005862 |
| 10 | Apocynin | C9H10O3 | 22.5 | 72.9928, 96.9597, 96.9691, 119.0501, 121.0292, 147.0447, 148.0484, 165.0549 | 165.0557 | 5.07 | NEG | 16687.88132 | 42143.65749 | 319983.4589 | 0.04342362 | 498-02-2 | Phenols | HMDB0247918 |
| 11 | Tilianin | C22H22O10 | 0 | No MS/MS spectrum detected | 447.1286 | 6.25 | POS | 0 | 3272.293502 | 12866.24265 | 0.001746024 | 4291-60-5 | Flavonoids | HMDB0302751 |
